# Emodin Attenuates the ECM Degradation and Oxidative Stress of Chondrocytes through the Nrf2/NQO1/HO-1 Pathway to Ameliorate Rat Osteoarthritis

**DOI:** 10.1155/2022/5581346

**Published:** 2022-01-17

**Authors:** Tianwen Ma, Yuanqiang Ma, Yue Yu, Lina Jia, Liangyu Lv, Xiaopeng Song, Jilang Tang, Xinyu Xu, Xuanbo Sheng, Ting Li, Li Gao

**Affiliations:** Heilongjiang Key Laboratory for Animal Disease Pathogenesis and Comparative Medicine, College of Veterinary Medicine, Northeast Agricultural University, Harbin 150030, China

## Abstract

Osteoarthritis (OA) substantially reduces the quality of life of the elderly. OA therapy remains a challenge since no treatment options for its causes are so far available. Over recent years, researchers have speculated that emodin may represent a potential treatment strategy for OA. However, it remains unclear whether the mechanism of action of emodin is associated with the inhibition of OA-induced oxidative stress. In the present study, the potential antioxidant mechanism of action of emodin and its protective properties against the development of OA were investigated both *in vitro* and *in vivo*. *In vitro*, emodin inhibited the production of reactive oxygen species (ROS) in chondrocytes induced by hydrogen peroxide (H_2_O_2_) and reduced the expression of matrix metalloproteinase (MMP)3 and MMP13 in a concentration-dependent manner. It was found that emodin upregulated the Nrf2/NQO1/HO-1 pathway, thereby attenuating the effects of oxidative stress caused by OA. In a rat model of posttraumatic OA induced by anterior cruciate ligament transection (ACLT), emodin reduced the extent of joint swelling. Emodin attenuated oxidative damage in the cartilage by upregulating superoxide dismutase (SOD), catalase (CAT), and glutathione (GSH) activity, reducing malondialdehyde (MDA) concentration and inhibiting the expression of the extracellular matrix (ECM) degradation biomarkers cartilage oligomeric matrix protein (COMP), and C-terminal telopeptide of type I collagen (CTX-I) and type II collagen (CTX-II), thereby reducing cartilage damage. In summary, the present study indicates that emodin reduces ECM degradation and oxidative stress in chondrocytes via the Nrf2/NQO1/HO-1 pathway, thereby ameliorating OA in rats.

## 1. Introduction

Osteoarthritis (OA) is the most common bone disease, causing joint pain or disability in the aged population (>65 years old), causing reduced quality of life and shortened life span [1]. Although many recent therapies alleviate OA, such as total joint replacement [2], disease-modifying OA drugs (DMOADs) [3], and targeted therapies for OA pain [4], treatments that reverse or prevent OA remain elusive. Thus, a novel treatment strategy that is effective and safe is urgently required.

The development of OA is significantly associated with oxidative stress due to reactive oxygen species (ROS) [5]. In normal conditions, articular chondrocytes are in a stable state, with no detectable mitotic activity. They are adapted to a low-oxygen environment in which the metabolism and synthesis of the extracellular matrix (ECM) are in dynamic balance [6]. In pathological conditions, such as OA, the oxygen tension in the synovial fluid fluctuates due to accelerated tissue metabolism, the phenomenon of ischemia-reperfusion, and continuous abnormal strain on the joints [7]. Excessive ROS functions as a secondary messenger that reduces ECM synthesis and induces chondrocyte apoptosis [8]. When cells are in a stable state, nuclear factor erythroid-2 related factor 2 (Nrf2) combines with the negative regulator Kelch-like epichlorohydrin-associated protein 1 (Keap1) in the cytoplasm to form a Keap1-Nrf2 dimer, which promotes the ubiquitination and degradation of Nrf2 [9]. Oxidative and electrophilic stress destroys key cysteine residues in Keap1 and inactivates the Keap1-cullin 3 ubiquitination system, allowing Nrf2 to accumulate in the cytoplasm that then ultimately transfers to the nucleus [10]. It then regulates the gene transcription of NAD(P)H:quinone oxidoreductase 1 (NQO1) and heme oxygenase 1 (HO-1), thereby protecting chondrocytes [11]. A large number of studies have shown that modulation of the Nrf2/NQO1/HO-1 pathway that inhibits oxidative stress is a potential strategy to treat the effects of OA [12–14].

Emodin (1,3,8-trihydroxy-6-methylanthraquinone) is a naturally occurring anthraquinone derivative and an active ingredient of Chinese herbs, including Polygonum cuspidatum, Rheum palmatum, Aloe vera, Polygonum multiflorum, and Cassia obtusifolia [15]. Emodin is also a potential protective agent for OA [16, 17], but its pharmacological mechanisms of action require a more detailed study. Emodin can promote the elimination of oxidation products in cells. It protects against synaptic impairment and oxidative stress induced by fluoride in SH-SY5Y cells by modulating the ERK1/2/Nrf2/HO-1 pathway [18] and against acute pancreatitis-associated lung injury by inhibiting NLPR3 inflammasome activation via the Nrf2/HO-1 signaling pathway [19]. However, it remains unknown whether the protective effect that emodin exerts on OA is associated with oxidative stress.

In the present study, rat chondrocytes were exposed to hydrogen peroxide to observe the antioxidation and antimatrix degradative effects of emodin via the Nrf2/NQO1/HO-1 signaling pathway. In addition, using a rat model of posttraumatic OA, the levels of antioxidant indicators (CAT, GSH, MDA, and SOD) and OA biomarkers (CTX-II, COMP, and CTX-1) were comprehensively analyzed after the administration of different concentrations of emodin to evaluate its potential mechanisms of protection against OA.

## 2. Materials and Methods

### 2.1. Isolation and Culture of Rat Primary Chondrocytes

Primary rat chondrocytes were cultured in accordance with a previously published protocol [20]. Briefly, the articular cartilage of the proximal tibia and distal femur was harvested from Sprague Dawley rats aged 14-21 days. After washing 3 times with sterile PBS (Solarbio, China), the articular cartilage was digested with 0.25% trypsin (Gibco, USA) for 30 min. Fresh cartilage samples were cut into small pieces of approximately 1 mm^3^ and digested with 0.2% type II collagen (Gibco) for 4-6 h, and then an equal volume of DMEM/F12 (Gibco) culture medium supplemented with 10% fetal bovine serum (FBS; Biological Industries, Israel) was added to terminate the digestion. After the chondrocytes had been transferred to a 15 ml tube and centrifuged, the supernatant was removed. The cells were seeded into a 25cm^2^ air-permeable culture flask containing DMEM/F12 supplemented with 10% FBS and 1% penicillin/streptomycin (Beyotime, China) then cultured at 37°C in a humidified atmosphere containing 5% CO_2_ at 37°C. Second-passage chondrocytes were used in subsequent experiments in the present study.

### 2.2. Identification of Rat Chondrocytes

Chondrocytes were identified in sections by immunofluorescent staining of type II collagen while proteoglycans secreted by chondrocytes were stained using toluidine blue. The chondrocytes were fixed in 4% paraformaldehyde (Beyotime) and stained by incubating in toluidine blue (Solarbio) solution prior to rinsing with deionized water to remove any impurities. The sections were sealed with neutral resin, then observed using an inverted microscope (Jiangnan Optoelectronics Co., Ltd., China).

The chondrocytes were seeded into 35 mm confocal dishes (Beyotime) and incubated until 80% confluent. The culture medium was discarded, and the cells blocked by covering evenly with 3% BSA (Beyotime) for 30 min at room temperature. Type II collagen antibody (Novus, USA) was added dropwise to each well then incubated in a humid environment overnight at 4°C. The culture plate was then placed on a decolorizing shaker and washed 3 times. After drying, anti-rabbit IgG (H+L), F(ab′)2 fragment (Alexa Fluor®488 conjugated; CST, USA), was added dropwise to cover the cultured cells. Nuclei were counter-stained with DAPI (Beyotime), and then, antifade polyvinylpyrrolidone mounting medium (Beyotime) was added dropwise to mount the slides after drying. The slides were then observed by confocal microscopy (Leica, Germany), and images were collected.

### 2.3. Cell Viability Assay

Emodin (purity ≥ 96%, CAS: 518-82-1) was purchased from TCI (Shanghai) Development Co., Ltd. (Shanghai, China). The cytotoxicity of emodin and H_2_O_2_ toward chondrocytes was measured using a cell counting kit-8 (CCK-8) assay (Beyotime) in accordance with the manufacturer's protocol. Briefly, first-passage chondrocytes were cultured for 24 h in a 96-well plate (5000/well) and then incubated with different concentrations of emodin (0, 5, 10, 15, and 20 *μ*M) or H_2_O_2_ (0, 0.1, 0.3, 0.4, 0.5, 1, and 2 mM) for 12 h. The cells were then washed with PBS, after which 100 *μ*l of DMEM/F12 supplemented with 10 *μ*l of CCK-8 solution was added to each well of the plate and incubated for an additional 30 min at 37°C. To establish the effective concentration of emodin required by chondrocytes to counteract the hydrogen peroxide with which they were stimulated; DMEM/F12 culture medium either with or without H_2_O_2_ was added to chondrocyte cultures and treated with different concentrations of emodin (0, 5, 10, and 20 *μ*M) for 12 h, after which the supernatant was combined with CCK-8 reagent and incubated together. The absorbance at 450 nm of each well was measured using a multifunctional microplate reader (BioTEK, USA). All measurements were performed in triplicate.

### 2.4. Measurement of ROS

ROS concentration was measured using dichlorodihydrofluorescein diacetate (DCFH-DA) staining (Beyotime). Chondrocytes (4 × 10^5^) were seeded in a 6 cm dish and treated with emodin and/or H_2_O_2_ for 12 h; then, intracellular ROS was measured after loading the cells with DCFH-DA for 30 min at 37°C, as described in a previous study [21, 22]. A fluorescence microscope was used to measure the fluorescent intensity of the DCF. Each experiment was conducted in triplicate.

### 2.5. Western Blotting

Total proteins were extracted from the chondrocytes using RIPA lysis buffer containing phenylmethanesulfonyl fluoride (Beyotime). Protein concentration was measured using a BCA protein assay kit (Thermo, USA). The proteins were separated using SDS-PAGE, after which the protein bands were transferred to PVDF membranes. After blocking with 5% skimmed milk, the membranes were incubated with a primary antibody against Nrf2 (1 : 2000, Affinity, China), HO-1 (1 : 1000, Wanleibio, China), NQO1 (1 : 2000, ABclonal, China), MMP3 (1 : 2000, ABclonal, China), MMP13 (1 : 2000, ABclonal, China), Lamin B1 (1 : 2000, Affinity, China), or GADPH (1 : 2000, ABclonal, China) overnight at 4°C. The membranes were subsequently incubated with a Rabbit Anti-Goat IgG (H+L) antibody (ZSGB-BIO, China) for 1 h at room temperature after which proteins were visualized using chemiluminescence. Each experiment was repeated three times. Use Tannon automatic gel image analysis system (Shanghai, China) to obtain protein bands. ImageJ software was used to analyze the relative expression level of the protein.

### 2.6. Quantitative Real-Time Polymerase Chain Reaction (qPCR) Analysis

The cells were treated with different concentrations of emodin (0, 5, 10, and 20 *μ*M) and H_2_O_2_ for 12 h. The total RNA of each group of cells was extracted using an RNAsimple total RNA kit (Tiangen, China). The RNA was reverse transcribed to complementary DNA (cDNA) using ReverTra Ace qPCR RT Master Mix with gDNA remover kit (Toyobo, Japan), and then, the expression of specific genes was quantified by q-PCR. SYBR Green SuperReal PreMix color was mixed with forward and reverse primers, cDNA, and ddH_2_O (Tiangen), and then 40 amplification cycles were performed. A LightCycler®480 (Roche, Germany) was used to monitor the fluorescence intensity during the entire PCR process to obtain the number of cycles until threshold (Ct) fluorescence had been achieved. Each experiment was repeated three times and performed in strict accordance with the manufacturer's instructions. The primer sequence is as follows: MMP3 (forward: 5′-TTTGGCCGTCTCTTCCATCC-3′; reverse: 5′-GCATCGATCTTCTGGACGGT-3′) and MMP13 (forward: 5′-TTCTGGTCTTCTGGCACACG-3′; reverse: 5′-TGGAGCTGCTTGTCCAGGT-3′).

### 2.7. Rat Model of Posttraumatic OA and Emodin Treatment

**Figure 1:**
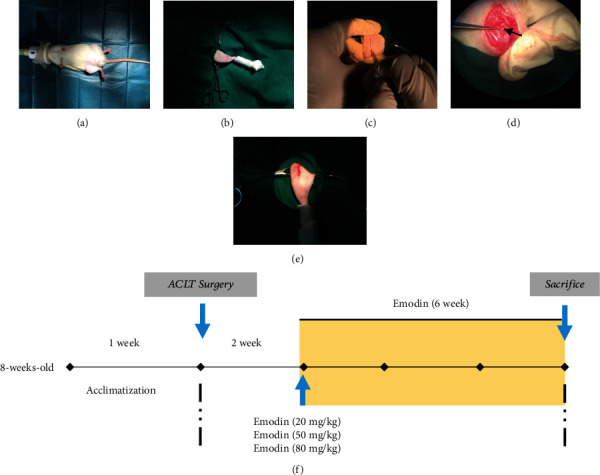
Establishment of rat posttraumatic OA model and *in vivo* experiment design. (a) Rats were anesthetized by inhalation of 2% isoflurane in oxygen/nitrous oxide. (b) Depilation and disinfection of the right knee joint of the rat. (c) Make a longitudinal 2-3 cm incision on the inside with a scalpel. (d) After the patella is displaced, the ACL is cut under a surgical microscope. (e) Under sterile conditions, use absorbable slits to suture the wound. (f) The flow chart of the in vivo study design, showing the time of administration of emodin, the treatment period, and the time of euthanasia.

A total of 40 male Sprague-Dawley rats (8 weeks, 240-320 g) were purchased from Changchun Yisi Experimental Animal Technology Co., Ltd. (Changchun, China), maintained within a controlled environment (light/darkness: 12/12 h; temperature: 23 ± 1°C) and allowed to move freely. Prior to surgery, the rats were acclimated for 1 week. In the present study, an anterior cruciate ligament transection (ACLT) model was established in which rats developed OA posttrauma, using a protocol described in a previous study [23] (Figures 1(a)–1(e)). The rats were randomly allocated into a control group (sham surgery), OA group (ACLT surgery that induced OA), 20 mg/kg emodin group, 50 mg/kg emodin group, or an 80 mg/kg emodin group, each group having 8 rats. Two weeks after ACLT surgery, 1 ml of emodin (20 mg/kg, 50 mg/kg, and 80 mg/kg) was administered to the rats by intraperitoneal injection once every two days (Figure 1(f)). The control group was injected with sterile saline. A suitable concentration of emodin was established based on a previous study [17]. After 6 weeks of administration of emodin, blood samples were collected from the rats through the tail vein. The blood was centrifuged at 1,000 g for 20 min at room temperature after which the supernatant was collected and stored at -80°C until required for additional analysis. Six weeks after administration of emodin or saline, the rats in all groups were euthanized by intraperitoneal injection of a lethal concentration of ether, and the tibia and femur were harvested. Every effort was made to minimize any animal suffering and reduce the number of animals required. The Laboratory Animal Welfare and Ethics Committee of Northeast Agricultural University approved the animal experiments and experimental design in the present study (#NEAU-2019-05-0254-7).

### 2.8. Knee Width Measurement

An electronic digital caliper was used to measure the width of each animal's knee joint to assess the extent of joint swelling [24]. Prior to ACLT surgery (0 weeks), the knee joint width was measured and then remeasured every two weeks until the end of the experiment 8 weeks after surgery. To reduce errors, measurement of knee joint width was performed by the same researcher. Each recorded value was the mean of three measurements during analysis.

### 2.9. Macroscopic Observation and Pelletier Score

The right knee joint of each rat was dissected and placed on ice to expose the tibial and femoral articular surfaces. Images of the tibial plateau and femoral condyle of all joints were acquired to assess cartilage damage (Nikon D5300 camera). The Pelletier scale (0-4 points) was used to score the morphology of the articular cartilage of the tibial plateau and femoral condyle [25]. Scoring was independently performed by two researchers blinded to the experimental details.

### 2.10. Safranin O Staining and Hematoxylin-Eosin (HE) Staining

Tibial and femoral samples from each rats were fixed in 10% neutral buffered formalin and then embedded in paraffin. The blocks were sliced into 5 *μ*m thick sagittal sections, and then, the cartilage matrix was stained with Safranin O while HE was used to evaluate the pathological changes to the cartilage. The Osteoarthritis Research Society International (OARSI) scoring system was used to evaluate the degree of knee cartilage pathological damage [26].

### 2.11. Antioxidant Activity Detection

A commercial kit was used to detect the activity of guaiacol peroxidase catalase (CAT) (Nanjing Jiancheng Bioengineering Institute, China), glutathione (GSH) (Nanjing Jiancheng Bioengineering Institute), and malondialdehyde (MDA) (Beyotime), superoxide dismutase (SOD) (Nanjing Jiancheng Bioengineering Institute) in rat serum. Strictly follow the kit manufacturer's instructions, and control the serum sample time within 5 min to reduce the error between each well. Three replicate wells are used for each set of samples. Use a multifunctional microplate reader to detect the OD value.

### 2.12. ELISA Detection of Rat OA Biomarkers

Serum levels of C-termianl telopeptide of type II collagen (CTX-II) (Mlbio, China), cartilage oligomeric matrix protein (COMP) (Nanjing Jiancheng Bioengineering Institute), and C-termianl telopeptide of type I collagen (CTX-I) (Nanjing Jiancheng Bioengineering Institute) were evaluated using commercially available Enzyme-linked immunosorbent assay (ELISA) kits according to the manufacturer's instructions. The ELISA kit is equilibrated at room temperature for 30 min, and the serum loading time is controlled within 5 min. Perform the ELISA procedure strictly in accordance with the manufacturer's instructions. Three replicate wells were used for each set of samples, and the OD value was averaged. Use a multifunctional microplate reader to detect the OD value. All experiments are conducted in the Heilongjiang Key Laboratory for Animal Disease Pathogenesis and Comparative Medicine.

### 2.13. Statistical Analysis

The SPSS 19.0 software (SPSS, Inc., Chicago, IL, USA) was used to analyze the experimental data. All data were expressed as the mean ± standard deviation (SD) and analyzed by a *t*-test. Multiple sets of data were analyzed by One-way ANOVA followed by Bonferroni post hoc test. A *P* < 0.05 was considered statistically significant.

## 3. Results

### 3.1. Chondrocyte Culture and Identification

**Figure 2:**
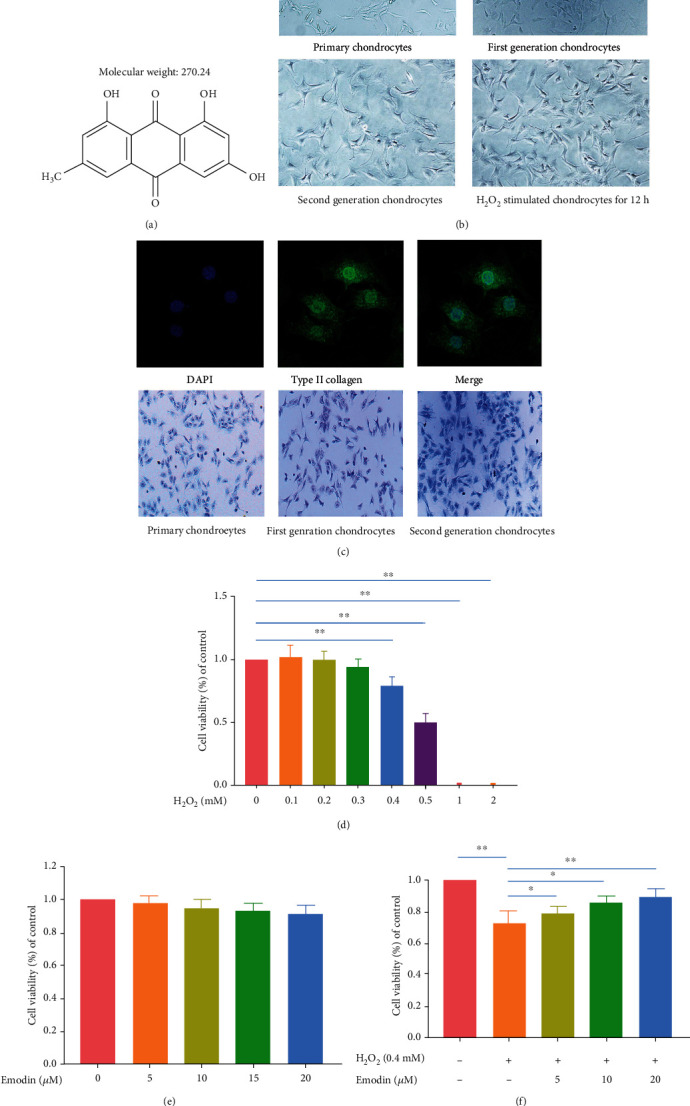
Primary chondrocytes and the effects of emodin on chondrocyte viability with and without H_2_O_2_. (a) Chemical structure of emodin. (b) Representative images of the morphology of primary rat chondrocytes, respectively, showing the changes in cell morphology and proliferation of primary chondrocytes, first-generation chondrocytes, second-generation chondrocytes, and H_2_O_2_ stimulation of chondrocytes for 12 h. (c) Representative image of type II collagen immunofluorescence and toluidine blue staining. Type II collagen in the ECM of chondrocytes is green fluorescent. (d) Use CCK-8 kit to detect the cell viability of chondrocytes treated with different concentrations of H_2_O_2_ (0, 0.1, 0.3, 0.4, 0.5, 1, and 2 mM) and (e) different concentrations of emodin (0, 5, 10, 15, and 20 *μ*M) for 12 h. (f) Adding 5, 10, and 20 *μ*M emodin to chondrocytes treated with 0.4 mM H_2_O_2_ can increase cell viability. The data were expressed as the means ± SD (*n* = 3). ^∗^*P* < 0.05 and^∗∗^*P* < 0.01.

The chemical structure of emodin is shown in Figure 2(a). Representative changes in morphology and proliferation of rat chondrocytes in different growth cycles are displayed in Figure 2(b). Second-passage chondrocytes exhibited an irregular polygonal morphology, with full nuclei and growing rapidly. After stimulation for 12 h with H_2_O_2_, the chondrocytes displayed a smaller, rounded morphology with a growth rate that had decreased significantly. The type II collagen in the ECM of the chondrocytes showed green immunofluorescence, and the proteoglycan was stained blue-violet by toluidine blue (Figure 2(c)).

### 3.2. Cell Viability

To initially determine the response of chondrocytes to treatment with H_2_O_2_, the cells were incubated for 12 h alone or in the presence of 0-2 mM H_2_O_2_ for 12 h. Cell viability was not affected by H_2_O_2_ at a concentration of 0.1-0.3 mM but displayed concentration-dependent cell death at higher levels (Figure 2(d)). After treatment with 0.4 mM H_2_O_2_, cell viability was approximately 80%, and so 0.4 mM H_2_O_2_ was selected to treat chondrocytes, to simulate the oxidative conditions found in OA. To determine whether emodin treatment affected cell viability, untreated chondrocytes were incubated for 12 h and compared with cells grown in the presence of 0-20 *μ*M emodin for 12 h (Figure 2(e)). We found that even at a concentration of 20 *μ*M, emodin did not affect cell viability. In addition, the addition of 5, 10, or 20 *μ*M emodin to chondrocytes treated with 0.4 mM H_2_O_2_ demonstrated an increase in cell viability (Figure 2(f)).

### 3.3. Emodin Inhibits H_2_O_2_-Induced ROS Generation in Chondrocytes

**Figure 3:**
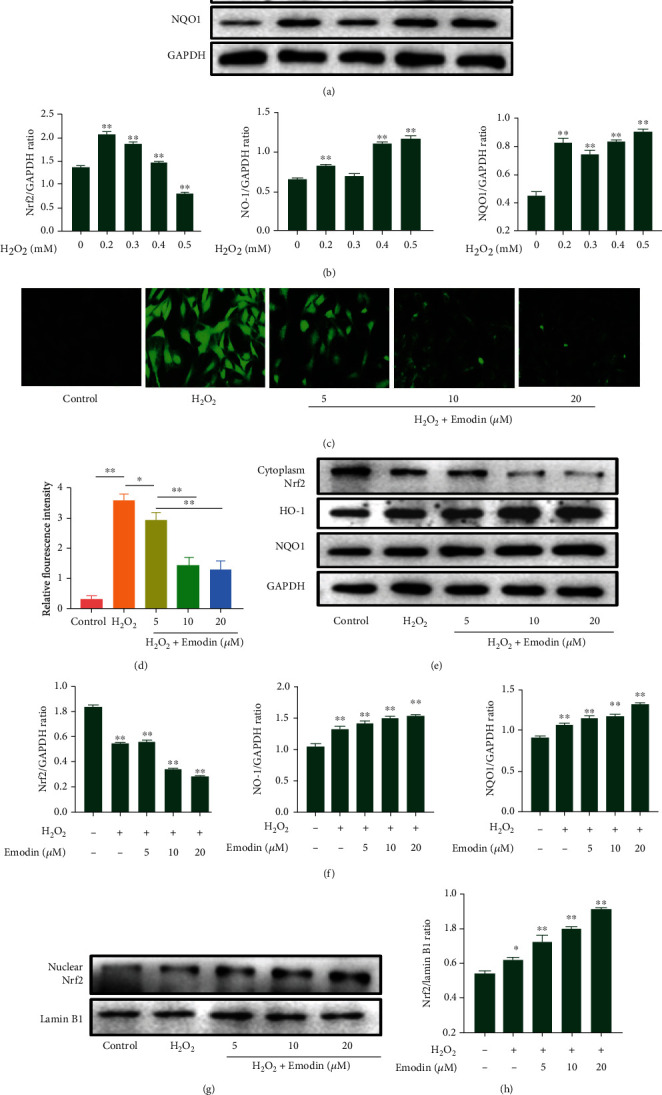
Effect of emodin on ROS and the Nrf2/NQO1/HO-1 signaling pathway in chondrocytes. (a, b) Western blot was used to detect the protein expression of Nrf2, HO-1, and NQO1 and to analyze the effect of different concentrations of H_2_O_2_ on oxidative stress of chondrocytes for 12 h. (c) DCFH-DA fluorescence staining demonstrated that emodin and H_2_O_2_ inhibited the overproduction of ROS when cocultured. (d) Mean fluorescence intensity of chondrocytes after administration of emodin. (e) Expression levels of cytosolic Nrf2, NQO1, and HO-1 evaluated by Western blots (f) were quantified. (g) Expression levels of nuclear Nrf2 evaluated by Western blots (h) were quantified. Data were expressed as the means ± SD (*n* = 3). ^∗^*P* < 0.05 and^∗∗^*P* < 0.01.

We speculate that the molecular mechanism of emodin attenuating oxidative stress activated by H_2_O_2_ may be related to the inhibition of ROS generation. Oxidative stress occurs in chondrocyte damage induced by H_2_O_2_ [27]. To test it, we conducted the following experiments under optimal conditions. We selected hydrogen peroxide 0, 0.2, 0.3, 0.4, and 0.5 mM to induce rat chondrocytes for 12 h, and Western blot detected the total Nrf2, HO-1, and NQO1 protein expression (Figures 3(a) and 3(b)), to determine the optimal concentration of H_2_O_2_ to induce oxidative stress in chondrocytes. We found that compared with the control group, the protein expression of Nrf2, HO-1, and NQO1 increased significantly at 0.4 mM H_2_O_2_ (*P* < 0.05). Interestingly, the concentration of Nrf2 decreased significantly at 0.5 mM H_2_O_2_ (*P* < 0.05). Therefore, we choose 0.4 mM H_2_O_2_ to induce cartilage for 12 h as an in vitro oxidative stress model.

Next, we aimed to determine whether emodin affects the overproduction of ROS when cocultured in H_2_O_2_, using DCFH-DA fluorescent staining. Compared with the control group, the green fluorescence intensity increased significantly after H_2_O_2_ treatment alone, and the ROS in chondrocytes decreased significantly after treatment with emodin (Figure 3(c)). In addition, emodin inhibited ROS levels in chondrocytes after H_2_O_2_ treatment in a concentration-dependent manner (*P* < 0.05) (Figure 3(d)).

### 3.4. Emodin Exerts an Anti-ECM Degradation Effect via by Activation of the Nrf2/NQO1/HO-1 Signaling Pathway in Chondrocytes

After treating chondrocytes induced by H_2_O_2_ with emodin, the expression of Nrf2 in the cytoplasm was reduced (Figures 3(e) and 3(f)) (*P* < 0.05), and the expression of Nrf2 in the nucleus was increased in a concentration-dependent manner (Figures 3(g) and 3(h)) (*P* < 0.05). To further clarify the mechanism of action of emodin against H_2_O_2_-induced chondrocyte protection, the protein expression levels of NQO1 and HO-1 were measured by Western blotting. Compared with control cells, the expressions of HO-1 and NQO1 in the H_2_O_2_ group were significantly increased (*P* < 0.05), while the expressions of HO-1 and NQO1 further increased after emodin treatment and increased in a concentration-dependent manner (Figures 3(e) and 3(f)) (*P* < 0.05).

**Figure 4:**
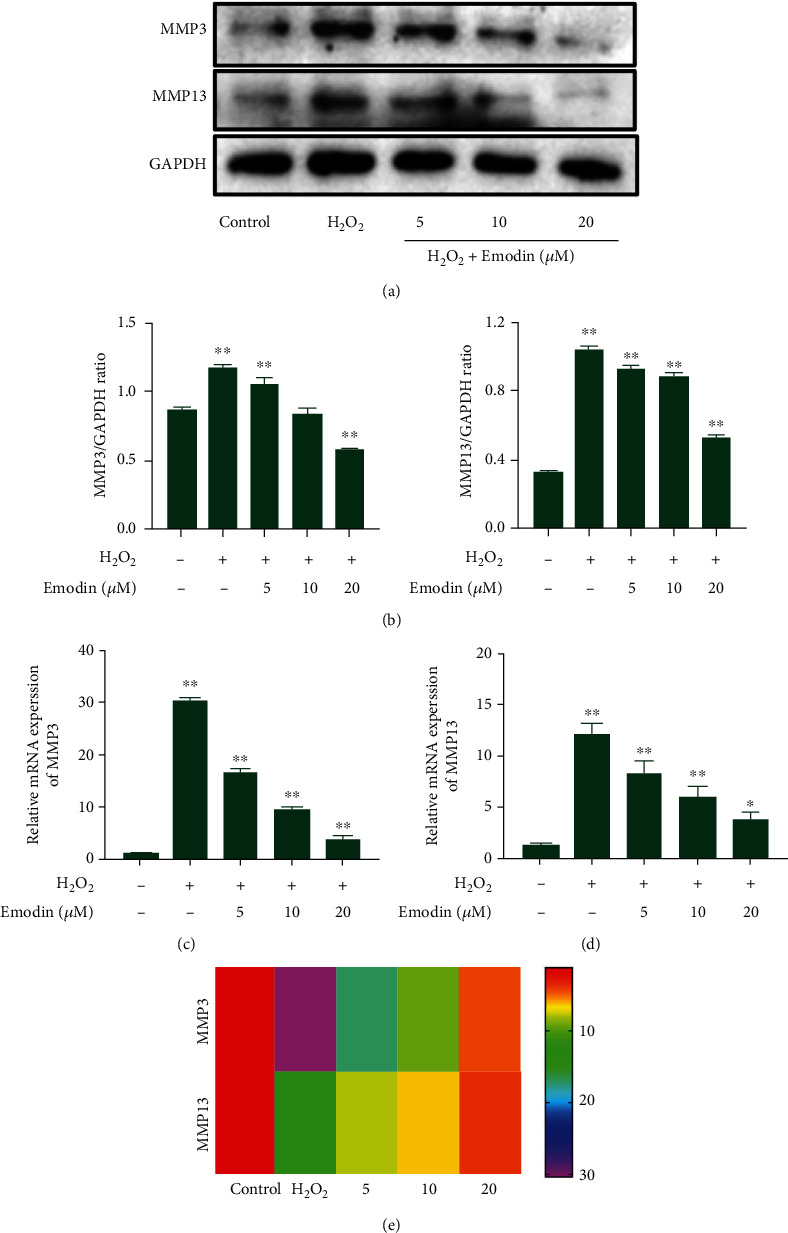
Effect of emodin on the mRNA and protein expression of MMP3 and MMP13 in chondrocytes induced by H_2_O_2_. (a) The protein expression levels of MMP3 and MMP13 were evaluated by Western blots and (b) quantified. (c) The mRNA expression levels of MMP3 and (d) MMP13 were evaluated by qPCR. (e) Heatmap of MMP3 and MMP13 expression in chondrocytes induced by H_2_O_2_. The data were expressed as the means ± SD (*n* = 3). ^∗^*P* < 0.05 and^∗∗^*P* < 0.01.

In addition, we tested the effect of emodin against ECM degradation and measured mRNA and protein expression levels of MMP3 and MMP13 in chondrocytes stimulated by H_2_O_2_ (Figure 4). In the emodin treatment group, compared with H_2_O_2_, the protein expression levels of MMP3 and MMP13 were significantly lower (*P* < 0.05) (Figures 4(a) and 4(b)). The mRNA expression of MMP3 and MMP13 was consistent with protein expression (Figures 4(c)–4(e)). In addition, emodin caused concentration-dependent inhibition of H_2_O_2_-induced ECM degradation in chondrocytes. The results above indicate that emodin exerts anti-ECM degradation properties by activation of the Nrf2/NQO1/HO-1 signaling transduction pathway in chondrocytes.

### 3.5. Emodin Reduces the Swelling of the Knee Joint in OA Rats

**Figure 5:**
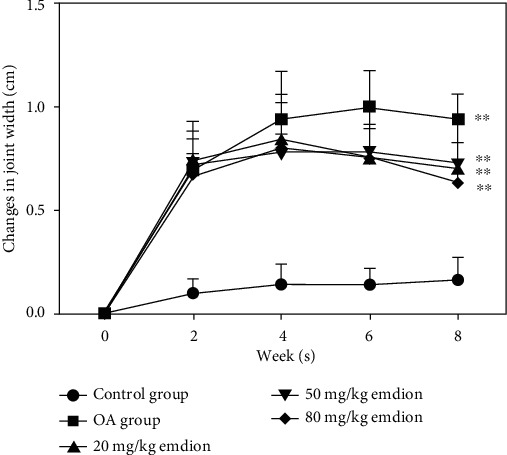
Effect of emodin on the swelling of the knee joint in rats. The data are presented as the means ± SD (*n* = 3). ^∗^*P* < 0.05 and^∗∗^*P* < 0.01 compared with the control group.

ACLT surgery induced posttraumatic OA in rats within 8 weeks. Compared with those in the control group, all rats in the OA group exhibited increased swelling due to joint instability. However, after treatment with emodin, the degree of swelling in the knee joints declined significantly. It is worth noting that after two weeks of administration, the effects of emodin were already apparent, with a concentration of 80 mg/kg having the greatest effect (*P* < 0.05) (Figure 5).

### 3.6. Macroscopic Observation of Tibial and Femoral Surfaces

**Figure 6:**
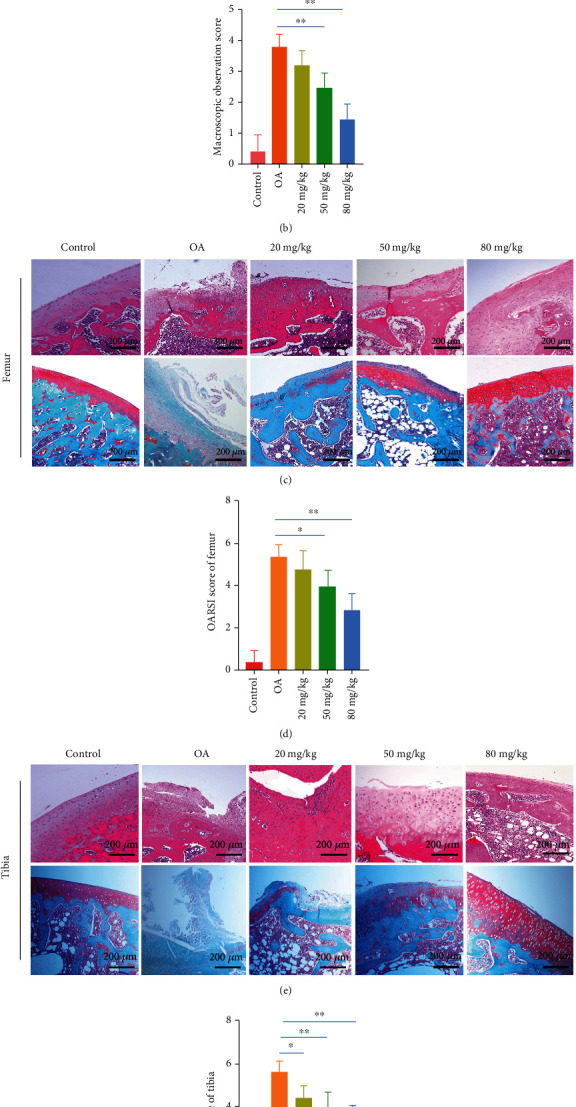
Progression of OA in rats induced by ACLT with or without emodin. (a) Macroscopic image and (b) Pelletier score of the rat knee joint 6 weeks after emodin administration. (c, e) The cartilage surface of the femoral condyle and tibial plateau of the control group was smooth without cartilage defects. Rats in the untreated OA group showed severe cartilage damage, the middle layer of cartilage was eroded, and the subchondral bone was exposed. Cartilage loss after emodin treatment is improved, and the degree of ulcers on the cartilage surface is reduced. After 6 weeks of emodin treatment, hematoxylin-eosin (HE) staining, Safranin O staining (scale bar: 200 *μ*m), and (d, f) OARSI score representative knee joint images. In the control group, chondrocytes had no vacuoles and Safranin O stained evenly. In the OA group, chondrocytes were severely damaged, chondrocytes were hypertrophy, and chondrocytes aggregated. After six weeks of treatment, emodin can improve the loss of chondrocytes and inhibit the loss of proteoglycan in ECM. The data are presented as the means ± SD (*n* = 3). ^∗^*P* < 0.05 and^∗∗^*P* < 0.01.

Changes to the surface of the cartilage in the femoral condyle in each group were observed macroscopically (Figure 6(a)). The cartilage surface of the knee joint in the control group was complete and shiny, without any visible damage to the cartilage surface. In the OA group, the surface of the cartilage was rough and dark red due to the cartilage surface and middle layer being corroded with severe ulcers. The cartilage surface had become thin with the subchondral bone partially exposed. After administration of different concentrations of emodin, damage to the surface of the cartilage gradually declined. Deep ulcers appeared on the surface of the femoral condyle in the 20 mg/kg emodin group, the surface of which was dark red with damage reaching to the middle of the cartilage. The tibial surface was rough and thin, although the surface of articular cartilage was intact. The surface layer of femoral condyle cartilage in the 50 mg/kg emodin group was slightly shiny, while damage to the cartilage layer was lower and joint instability was caused by local ulcers and thinning of the cartilage layer at the edge of the femur. In the 80 mg/kg emodin group, the surface of the tibial plateau and femoral condyle had recovered their luster. The surface of the femur was slightly rough and the surface of the tibia had eroded, with erosion ulcer marks on the edge. Macroscopically, cartilage surface scores of the knee joints of rats were significantly lower in the 50 mg/kg and 80 mg/kg emodin groups compared with those of the OA group (*P* < 0.05) (Figure 6(b)).

### 3.7. Pathological Progression of OA in Rats Induced by ACLT with or without Emodin

We further examined pathological changes to the cartilage of the tibia and femur due to emodin in ACLT-induced OA to confirm whether the results obtained by macroscopic observation were consistent with histopathological observation (Figures 6(c) and 6(e)). The surface of the tibial cartilage in the control group was smooth, with cartilage structure and layers that were clearly defined. The chondrocytes were arranged neatly, without vacuolization or cell aggregation, and without inflammatory cell infiltration. In the OA group, the chondrocytes were severely damaged, the surface of the articular cartilage was destroyed, and the number of cells reduced. The chondrocytes were hypertrophic and clustered, indicating that OA had progressed significantly. After six weeks of experimental treatment with emodin, HE staining revealed that it has ameliorated the loss of cartilage cells, stabilized the microenvironment of the cartilage, and reduced the progression of arthritis. Histological staining for proteoglycans in the ECM of the chondrocytes was also examined. As the concentration of emodin increased, the surface of the joints became smoother, and OARSI scores gradually decreased (Figures 6(d) and 6(f)), with positive Safranin O staining, indicating that the degradation of the ECM gradually declined. It is worth noting that the OARSI scores of the 50 mg/kg and 80 mg/kg emodin groups were significantly lower than that of the OA group, with Safranin O staining intensity of the 80 mg/kg emodin group similar to that of the control group.

### 3.8. Preventive Action of Emodin on Oxidative Stress in ACLT-Induced Rats

**Figure 7:**
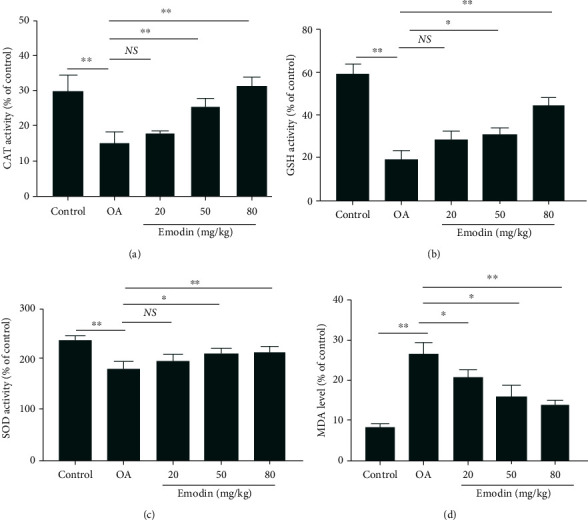
Effects of emodin on the levels of CAT, GSH, SOD, and MDA in the serum of OA rats. Data were expressed as the means ± SD (*n* = 3). ^∗^*P* < 0.05 and^∗∗^*P* < 0.01. NS: no significant difference.

Figure 7 displays the preventive effect of emodin on oxidative stress in OA rats. Compared with the control group, the activity of the antioxidant enzymes CAT, GSH, and SOD in the serum of OA rats induced by ACLT was significantly lower (Figures 7(a)–7(c)), the concentration of MDA significantly higher (Figure 7(d)), and the extent of lipid peroxidation significantly greater (*P* < 0.05). Compared with the OA group, as the concentration of emodin increased, lipid peroxidation decreased significantly, serum SOD, CAT, and GSH activity gradually increased, and serum MDA levels decreased significantly (*P* < 0.05). The 50 mg/kg and 80 mg/kg emodin concentrations were the most effective (*P* < 0.05). Emodin was found to significantly inhibit lipid peroxidation in the *in vivo* model of OA and increase the activity of the antioxidant enzymes SOD, CAT, and GSH.

### 3.9. Emodin Inhibits the Level of Cartilage/Bone Biomarkers in ACLT-Induced Rats

**Figure 8:**
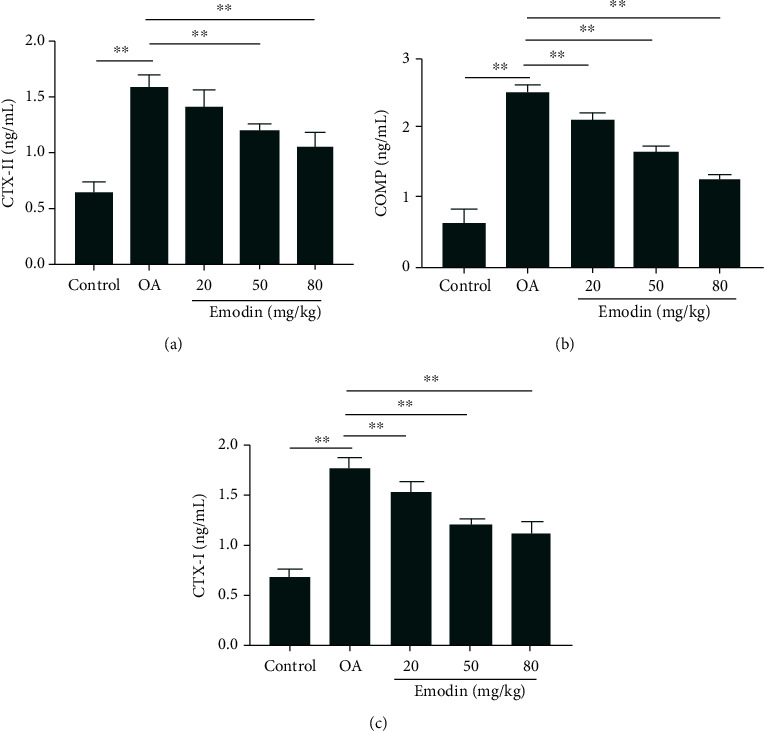
Effects of emodin on the levels of CTX-II, COMP, and CTX-I in the serum of OA rats. The data were expressed as the means ± SD (*n* = 3). ^∗^*P* < 0.05 and^∗∗^*P* < 0.01.

To establish the protective effects of emodin on the joints of animals with OA, the levels of cartilage degradation markers CTX-II and COMP (Figures 8(a) and 8(b)) and the bone turnover marker CTX-I (Figure 8(c)) were measured in serum. The results indicate that compared with the control group, serum CTX-II, COMP, and CTX-I levels increased significantly eight weeks after ACLT surgery (*P* < 0.05). Compared with the OA group, serum CTX-II, COMP, and CTX-I levels were significantly lower after administration of emodin (*P* < 0.05). In addition, emodin reduced the levels of the cartilage degradation markers CTX-II and COMP and the bone turnover marker CTX-I in a concentration-dependent manner.

## 4. Discussion

Chinese and garden rhubarb are “generally recognized as safe” (GRAS) as a food [28]. Emodin is a polyphenol (anthraquinone) compound isolated from the roots and rhizomes of *Rheum palmatum* [29]. Over recent years, the mechanism by which emodin prevents and treats OA has been extensively explored. Emodin promotes the proliferation of chondrocytes and downregulates the expression of inflammatory mediators in chondrocytes by inhibition of the ERK and Wnt/*β*-catenin pathways [30]. By downregulation of IL-1*β* that induces chondrocyte NF-*κ*B and Wnt/*β*-catenin pathways, emodin inhibits the expression of MMP13, ADAMTS-4, and ADAMTS-5 [16]. Emodin reduces apoptosis in ATDC5 cells induced by LPS and inhibits the Notch and NF-*κ*B pathways by upregulation of TUG1 that exerts anti-inflammatory effects [31]. Emodin is effective in inhibiting IL-1*β*-induced chondrocyte production of inflammatory factors such as iNOS, NO, COX-2, and PGE2 [17]. Liu et al. demonstrated that emodin reduces the cytotoxicity of IL-1*β* toward rat chondrocytes in a concentration-dependent manner [30]. In a previous study, emodin was investigated for the treatment of OA based on its anti-inflammatory and antiapoptotic properties. In the present study, we studied the effects of emodin against oxidative stress levels in OA. The results indicate that emodin activates the Nrf2/NQO1/HO-1 pathway in a concentration-dependent manner, thereby reducing H_2_O_2_-induced ROS production and oxidative stress. We speculate that rhubarb inhibits ECM degradation via the Nrf2/NQO1/HO-1 pathway and also plays a potential antioxidant role in the prevention and treatment of OA and could possibly represent a preventive supplement for human OA.

Elevated levels of ROS in cells indicate insufficient antioxidant capacity leading to oxidative stress. Excessive H_2_O_2_ is considered the key mediator of damage to cells (including chondrocytes) [32]. A particular concentration of H_2_O_2_ inhibits the synthesis of chondrocyte ECM, causing chondrocyte apoptosis, lipid peroxidation, and excessive release of proinflammatory cytokines, leading to the formation of MMPs and ADAMTS, and directly resulting in articular cartilage damage [33, 34]. Gao et al. used H_2_O_2_ to induce chondrocyte damage and establish an OA oxidative stress model in rat tissue *in vitro*, finding that H_2_O_2_ induced ROS production in chondrocytes [35]. We speculate that the increase in Nrf2 expression caused by 0.4 mM H_2_O_2_ may represent a self-adaptive mechanism for chondrocytes to survive in a pathological microenvironment characterized by increased levels of inflammatory molecules and oxidative stress [36, 37]. When the concentration of H_2_O_2_ reaches 0.5 mM, the level of ROS in chondrocytes increases and the expression of Nrf2 decreases. This imbalance may be due to the increased oxidative stress of chondrocytes, while the endogenous antioxidant defense system in the chondrocytes does not adequately improve oxidative damage [38]. In addition, we used 0.4 mM H_2_O_2_ to induce chondrocyte injury and found that emodin reduced the cytotoxicity of H_2_O_2_ in rat chondrocytes in a concentration-dependent manner. After H_2_O_2_ stimulation, the continuous expression of HO-1 andNQO1 indicates that the body has continuous resistance to chondrocyte oxidative damage [39] and also proves that the *in vitro* oxidative stress model was successfully induced. Therefore, we chose 0.4 mM H_2_O_2_ to induce 12 h to simulate the oxidative damage of chondrocytes *in vitro*.

Increasing numbers of studies have shown that herbs and dietary supplements exert antioxidant effects via the Nrf2/NQO1/HO-1 signaling pathway [12, 14, 40], which plays a key role in preventing oxidative damage by regulation of intracellular redox homeostasis. Antioxidant capacity is also closely related to the expression levels of antioxidant enzymes regulated by Nrf2 [41]. After chondrocytes experience oxidative stress, the conformation of Keap1 changes, activating Nrf2 bound to Keap1 in the cytoplasm and dissociating into the nucleus [5]. Nuclear Nrf2 activates the expression of HO-1, NQO1, SOD, GPX, and CAT antioxidant protease [42, 43]. When chondrocytes are stimulated by H_2_O_2_, the intracellular balance is broken, and the levels of antioxidant proteases in chondrocytes are then not sufficient to remove large quantities of oxidation products [44]. After treatment with emodin, the expression of Nrf2 in the nuclei of chondrocytes increased significantly in a concentration-dependent manner, and levels of Nrf2 in the cytoplasm decreased significantly. This indicates that emodin promotes the transfer of Nrf2 into the nucleus and represents a key mechanism by which cell oxidative damage is reduced. In addition, emodin increased the expression of HO-1 and NQO1 proteins in damaged chondrocytes, indicating that the concentration of antioxidant proteases in the cells increased, which reduces the degree of cell damage. This was attributed to emodin which promotes the accumulation of Nrf2 in the nucleus and activates the expression of downstream antioxidant enzyme genes. We selected SOD, GPX, CAT, and MDA as indicators of oxidative stress in *in vivo* experiments. The present research indicates that the activity of CAT, GSH, and SOD reduces significantly after induction OA in the ACLT model, while MDA levels increase, consistent with the results of previous studies [45–47]. After the administration of emodin, CAT, GSH, and SOD activity in the serum of rats increased significantly. This indicates that GSH-peroxidase increases the oxidation of GSH and SOD catalyzes the disproportionation of superoxide anion free radicals to form oxygen and hydrogen peroxide, while CAT accelerates the decomposition of hydrogen peroxide, working together to inhibit the ROS produced by cartilage damage. Emodin attenuates the level of MDA in the serum of ACLT rats, indicating that emodin can inhibit lipid peroxidation in rat cartilage tissue. The inhibition of lipid peroxidation may be due to the free radical scavenging activity of emodin, further confirming its antioxidant capacity. Whether emodin acts purely as a free radical scavenger or through the stimulation of antioxidant enzyme activity necessitates further investigation.

ACLT surgery destabilizes the knee joints of rats, increasing friction between cartilage layers, causing mechanical damage to the articular cartilage. The increase in subchondral bone resorption and formation is an important feature of the ACLT model [48]. Both macroscopic observation and histopathological staining in the present study confirmed that joint damage in the rat following ACLT surgery caused a serious imbalance in the redox reaction of articular cartilage, with the cartilage/bone biomarkers CTX-II, COMP, and CTX-1 and abnormal metabolism verifying the successful establishment of a posttraumatic OA model. After the administration of emodin, the integrity of the cartilage surface gradually recovered, and the number of chondrocytes gradually increased. These results demonstrate that as the concentration of emodin increased, cartilage damage gradually declined, with increased Safranin O staining, inhibition of cartilage ECM degradation, reduced cell damage, and significantly increased MDA content. We found that the protein and mRNA expression levels of MMP3 and MMP13 in chondrocytes stimulated by H_2_O_2_ decreased significantly as the concentration of emodin increased. This demonstrates that emodin inhibits the expression of matrix-degrading enzymes in chondrocytes both *in vivo* and *in vitro*, and so it has been fully confirmed that emodin can inhibit chondrocyte damage, slowing the degradation of ECM, having a protective effect on joints.

OA biomarkers are widely used in OA diagnosis, to determine the stage of OA disease or to evaluate the safety and effectiveness of new drugs or therapies [49]. CTX-II and COMP can provide an estimate of the extent of cartilage ECM degradation. Isorhamnetin inhibits the expression of COMP and CTX-II in OA rats induced by MIA and prevents cartilage damage [50]. CTX-I in serum reflects the degradation of type I collagen in bone tissue [51]. A novel peracetylated oleuropein derivative was found to reduce serum COMP levels in a murine collagen-induced arthritis model [52]. Bone and cartilage degradation, as assessed by CTX-I and CTX-II plasma levels, decreased in all KBP-treatment groups while KBP potently inhibited bone resorption *in vitro* [53]. Both clinical studies on OA patients [54] and animal models [55, 56] of OA have shown increased levels of CTX-1. In the present study, CTX-1 levels were found to increase following ACLT, consistent with the results of Mohan et al. [56]. CTX-II and COMP levels in the present study in the serum of rats in the model group were significantly higher than those in the control group, indicating that type II collagen and proteoglycans in cartilage ECM in rats with knee joint injury were degrading. After the administration of emodin, the levels of CTX-II and COMP in rat serum decreased significantly, indicating that emodin inhibits ECM degradation and exerts a chondroprotective effect. Interestingly, we found that with increased emodin concentration, the CTX-I levels in the serum of rats gradually decreased. We speculate that emodin not only inhibits the degradation of ECM in cartilage but also regulates the metabolic processes of subchondral bone, increasing bone density and bone load capacity, although the specific mechanisms of action require additional study.

## 5. Conclusion

**Figure 9:**
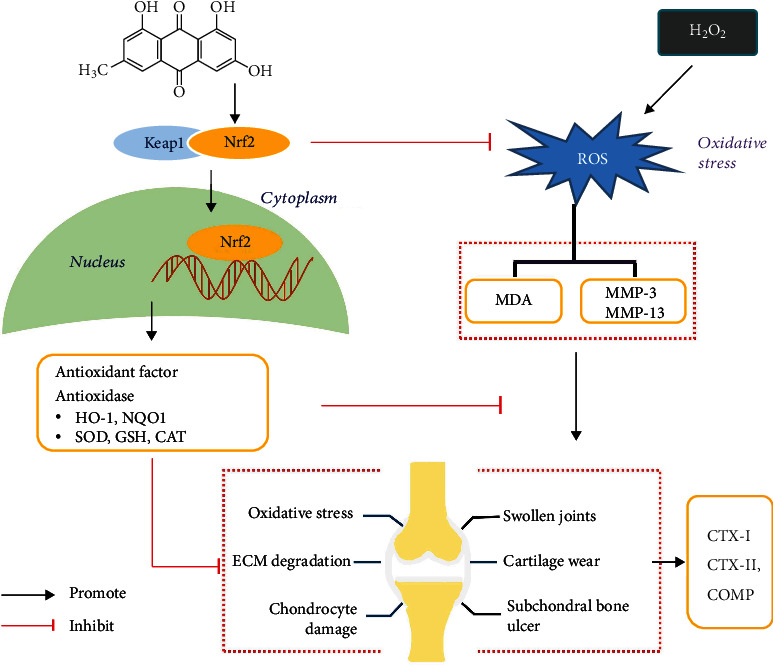
Emodin activates Nrf2/NQO1/HO-1 signaling which inhibits oxidative stress and ECM degradation in OA.

We identified that emodin can activate the Nrf2/NQO1/HO-1 pathway, thereby reducing the expression of MMP3, MMP13, and MDA, and promoting antioxidant enzymes, inhibiting the expression of OA biomarkers, reducing the damage caused by cartilage ECM and protecting joints (Figure 9). These findings provide novel insight into the mechanisms by which emodin ameliorates OA.

## Data Availability

The data used to support the findings of this study are included within the article.

## Supplementary Materials

Supplementary Materials The supplementary material provides the antibody manufacturers used in the Western blot test and all complete blots with molecular weight markers.
