# Supplementary material for: Emodin Attenuates the ECM Degradation and Oxidative Stress of Chondrocytes through the Nrf2/NQO1/HO-1 Pathway to Ameliorate Rat Osteoarthritis
Source: Oxid Med Cell Longev. 2022 Jan 17;2022:5581346. doi: 10.1155/2022/5581346 (PMC11427723; doi:10.1155/2022/5581346)
Supplement: Supplementary Materials — The supplementary material provides the antibody manufacturers used in the Western blot test and all complete blots with molecular weight markers. [file 5581346.f1.docx]

**The supplementary material provides the antibody manufacturers used in the Western blot test and all complete blots with molecular weight markers.**

Reagent

1. Marker (Catalog No.MA0342, meilunbio, China)


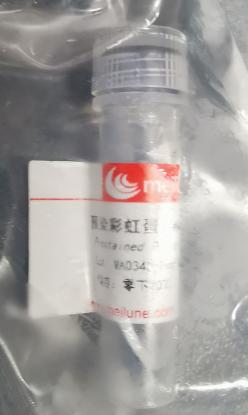

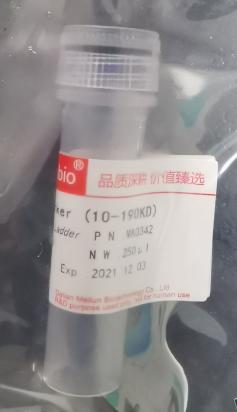

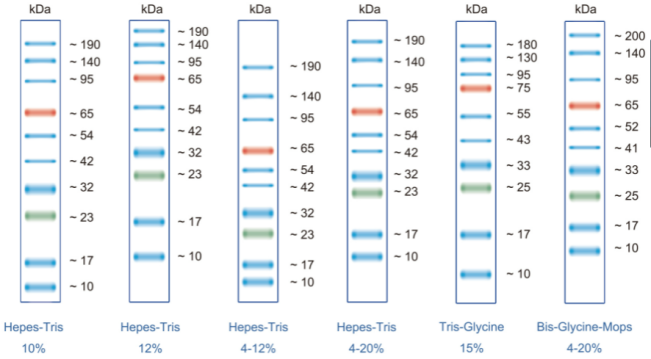


1. Nrf2 Antibody (Catalog No.AF0639, Affinity, China) (1:2000) (68kDa and 100kDa)


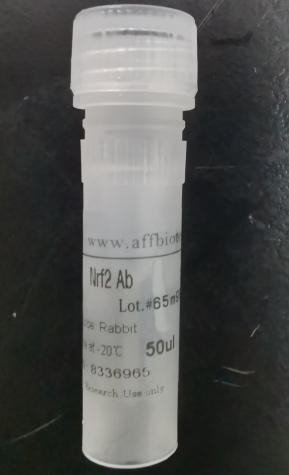

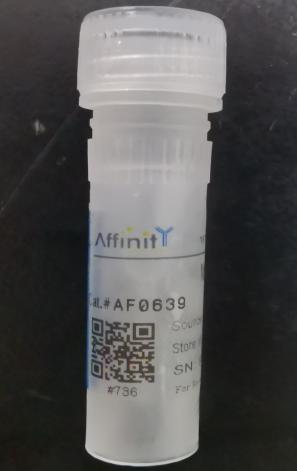


1. HO-1/Heme Oxygenase 1Antibody (Catalog No.WL02400, Wanleibio, China) (1:1000) (32kDa)


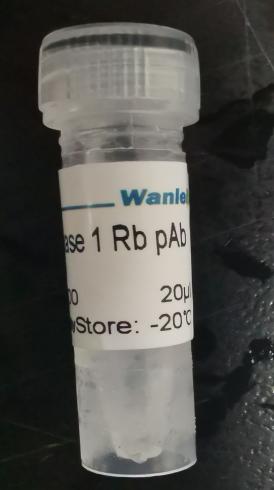

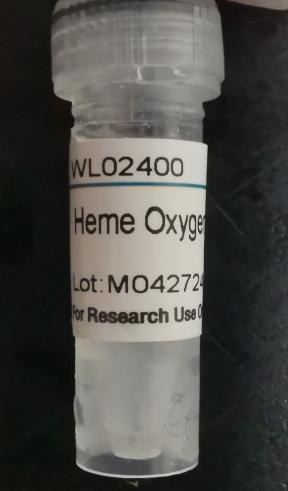


1. NQO1 Antibody (Catalog No.A19586, ABclonal, China) (1:2000)(31kDa)


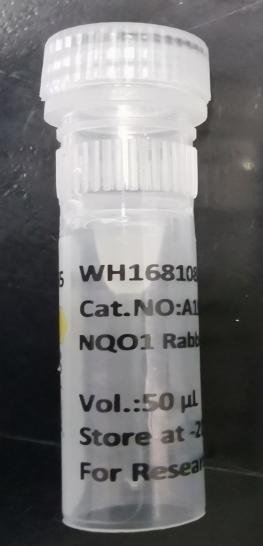

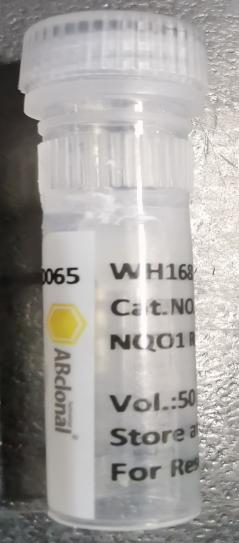


1. MMP3 Antibody (Catalog No.A11418, ABclonal, China) (1:2000)(60kDa)


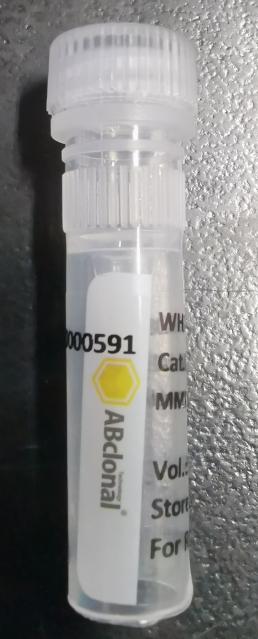

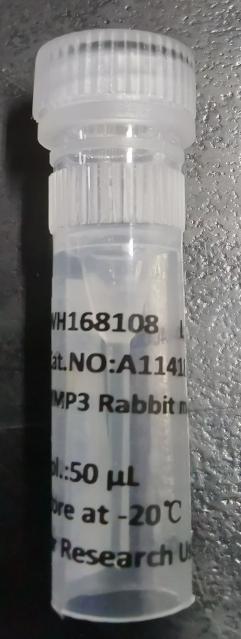


1. MMP13 Antibody (Catalog No.A11148, ABclonal, China) (1:2000)(60kDa)


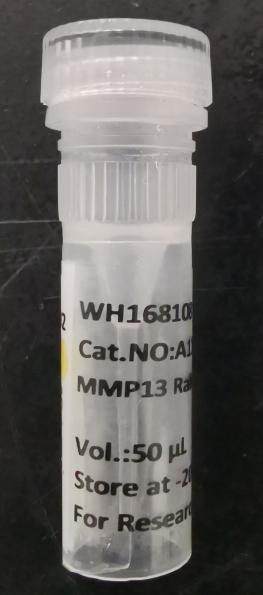

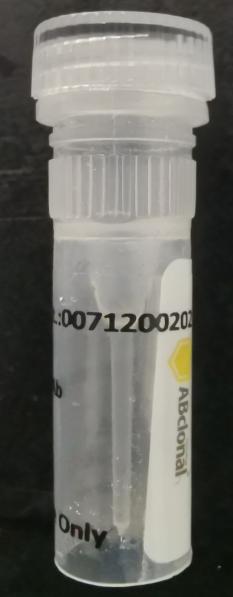


1. GAPDH Antibody (Catalog No.A11148, ABclonal, China) (1:2000)(36kDa)


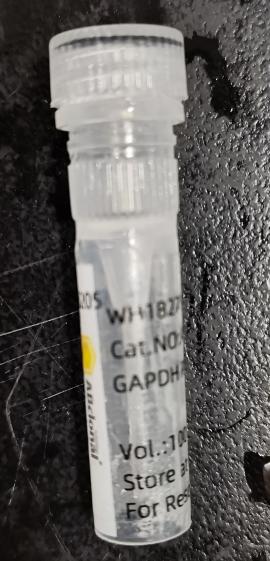

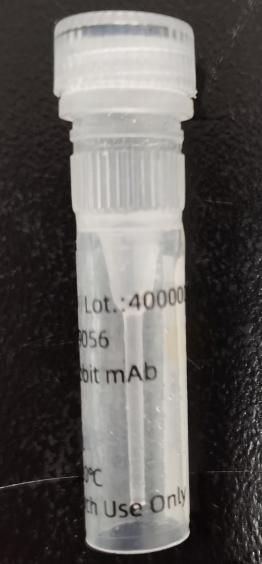


1. [Rabbit Anti-Goat IgG (H+L)](http://www.affbiotech.cn/goods-15080-S0010-Rabbit+Anti-Goat+IgG+(H_L)+HRP.html) (Catalog No.ZB-2306, ZSGB-BIO, China) (1:3000)


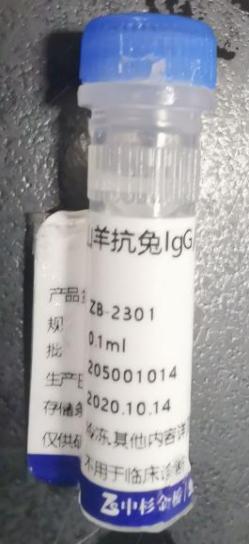

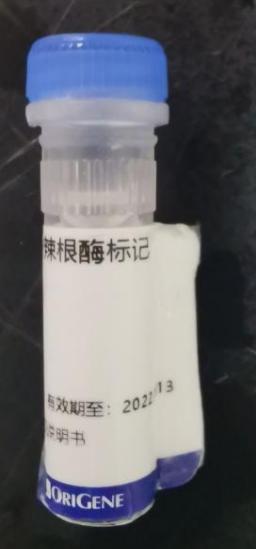


1. Lamin B1 Antibody (Catalog No.AF5161, Affinity, China) (1:2000) (66kDa)


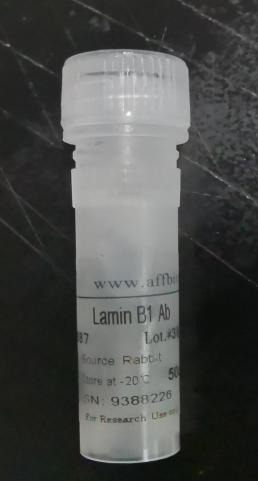

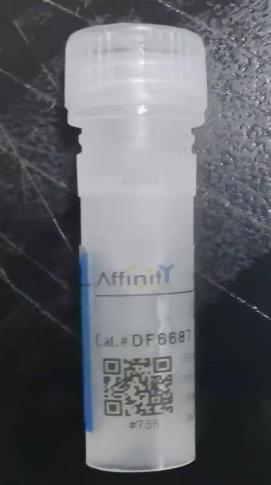


Figure 3a

1.HO-1(32kDa)

H_2_O_2_ (mM) 0 0.2 0.3 0.4 0.5


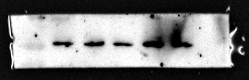

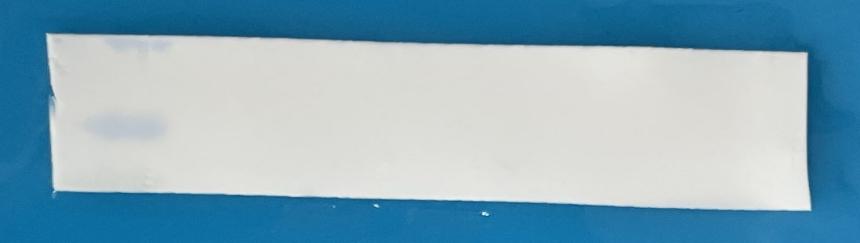

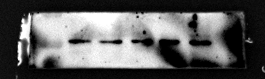

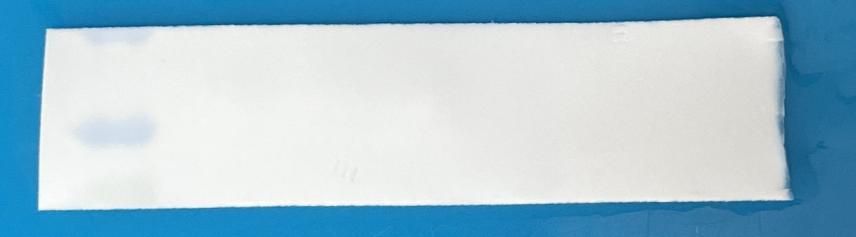

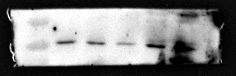

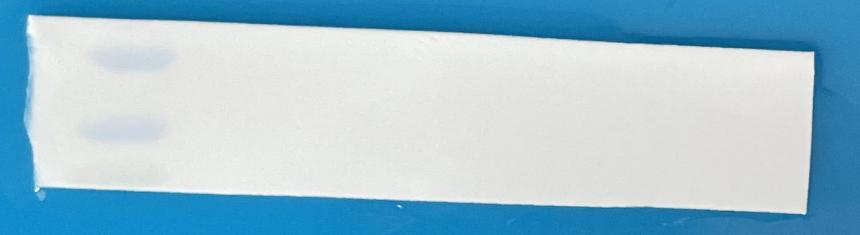


3

2

1

2.NQO1(31kDa)

H_2_O_2_ (mM) 0 0.2 0.3 0.4 0.5

1

2

3


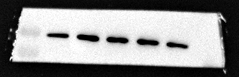

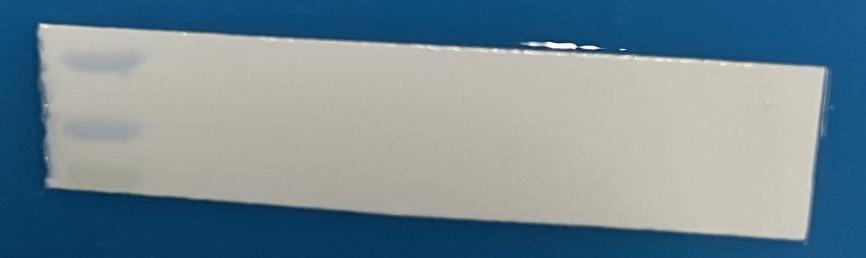

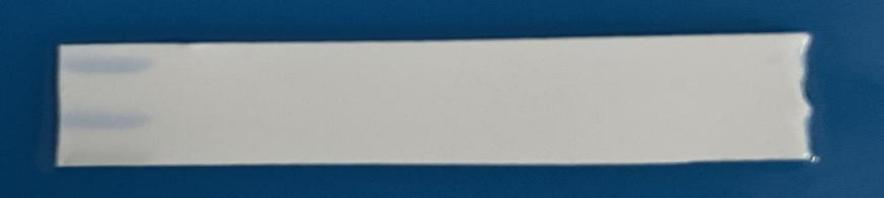

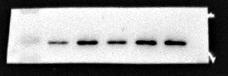

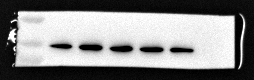

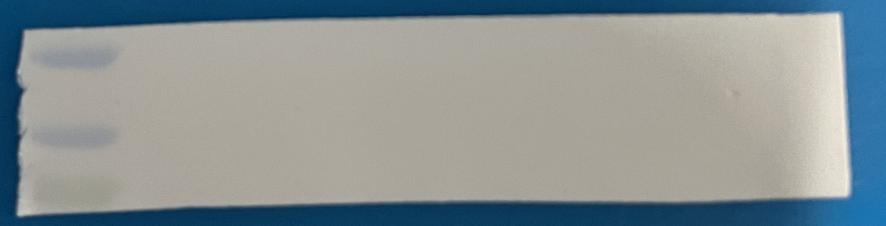


3

3.Nrf2 [Reference value provided by the manufacturer 100kDa; 68kDa (Calculated)].

H_2_O_2_ (mM) 0 0.2 0.3 0.4 0.5


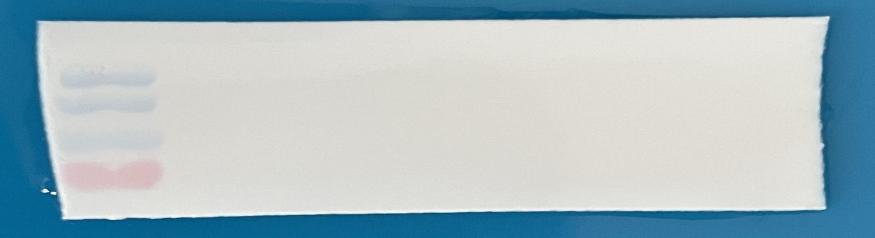

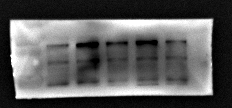

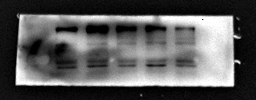

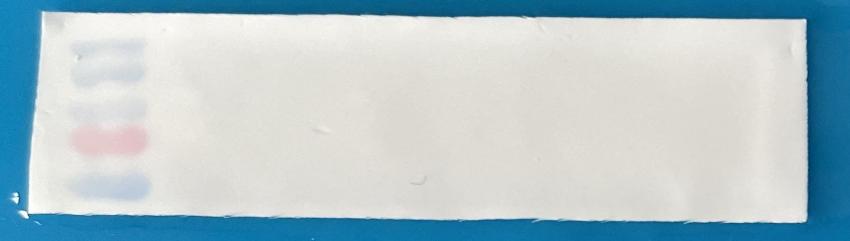

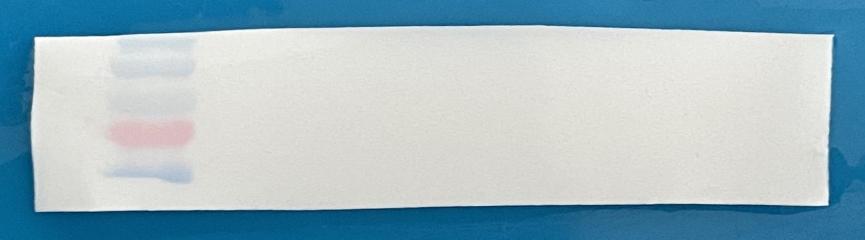

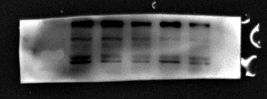


3

2

1

Figure 3e

4.HO-1(32kDa）

_E_modin(μM) _- - 5 10 20_

H_2_O_2_ (mM) _- + + + +_


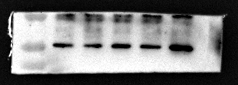

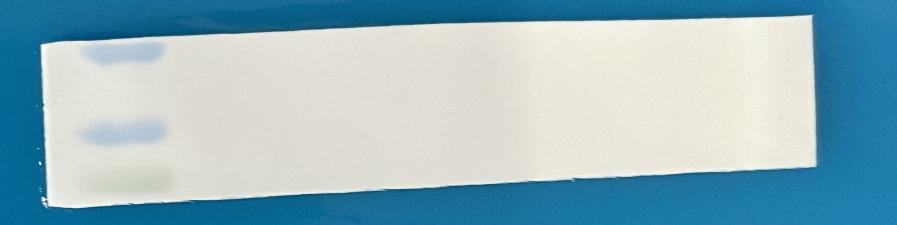

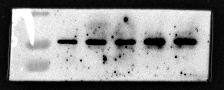

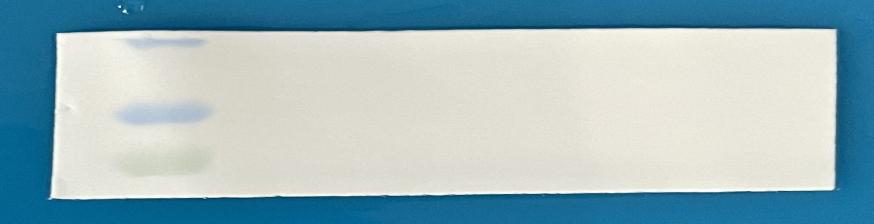

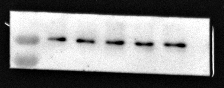

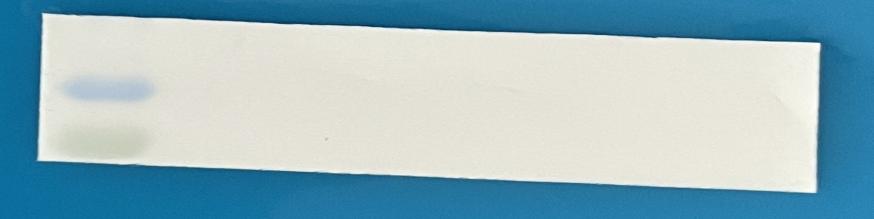


3

2

1

5.NQO1(31kDa）

_E_modin(μM) _- - 5 10 20_

H_2_O_2_ (mM) _- + + + +_

1

3

2


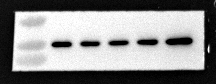

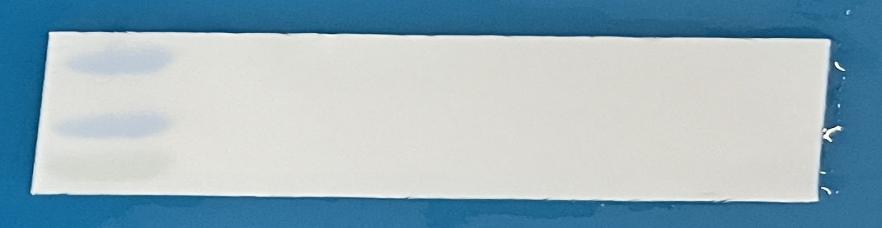

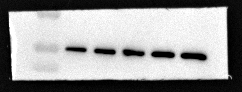

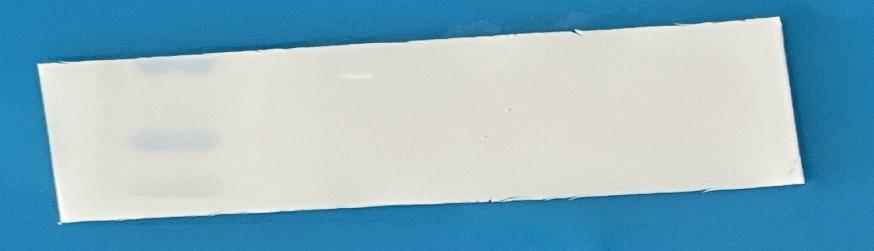

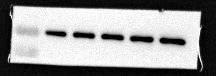

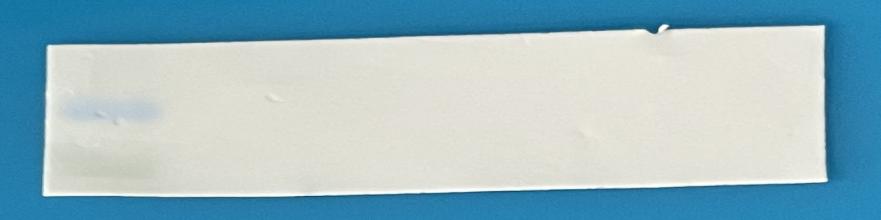


6.Nrf2 [Reference value provided by the manufacturer 100kDa; 68kDa (Calculated)] (Cytoplasm)

_E_modin(μM) _- - 5 10 20_

H_2_O_2_ (mM) _- + + + +_


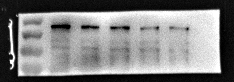

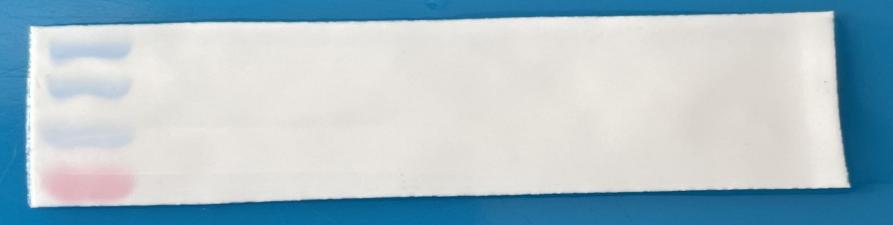

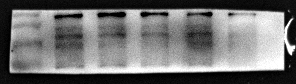

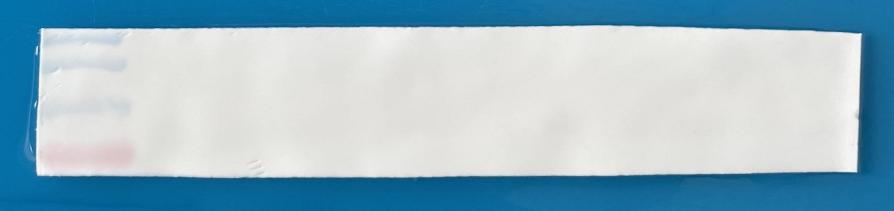

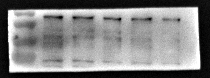

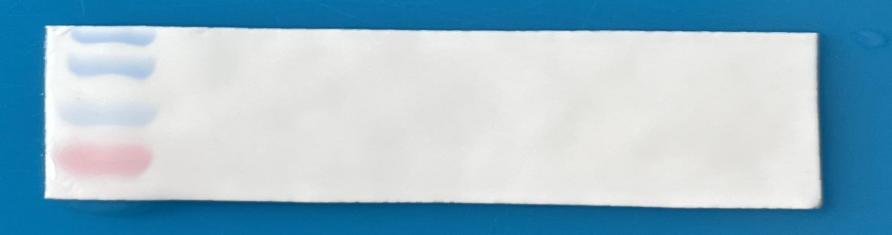


Figure 3g

7.Nrf2 [Reference value provided by the manufacturer 100kDa; 68kDa (Calculated)] (Nucleus)

_E_modin(μM) _- - 5 10 20_

H_2_O_2_ (mM) _- + + + +_

3

2

1


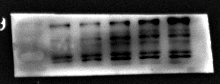

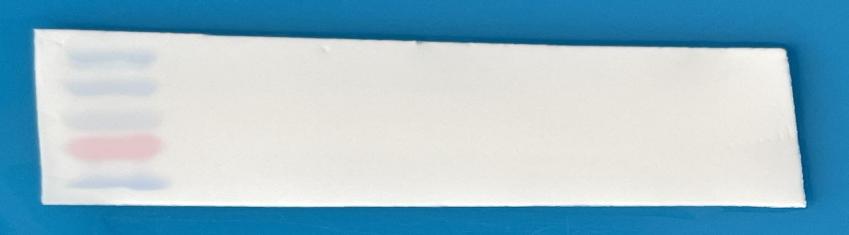

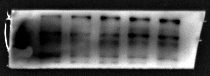

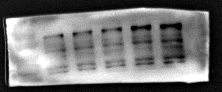

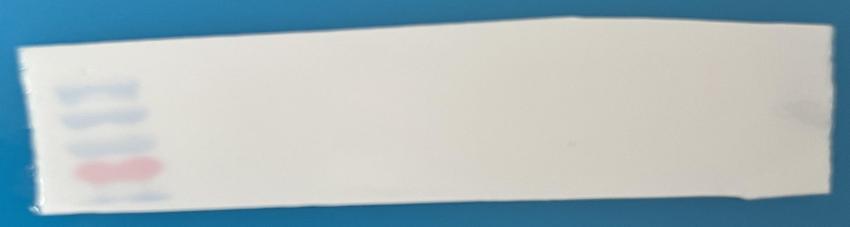

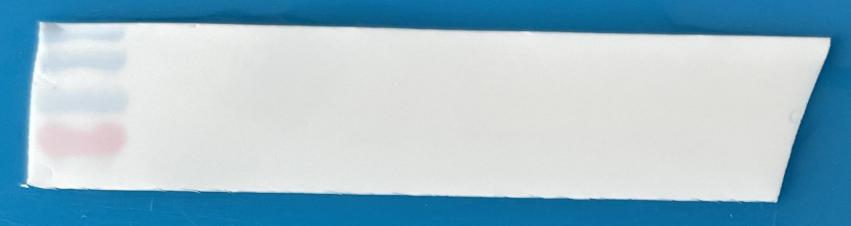


Figure 4a

8.MMP3 (60kDa）

_E_modin(μM) _- - 5 10 20_

H_2_O_2_ (mM) _- + + + +_


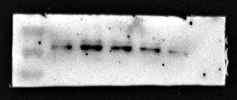

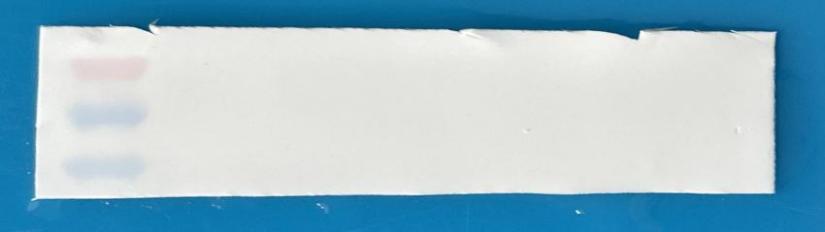

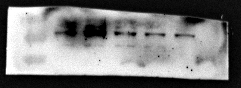

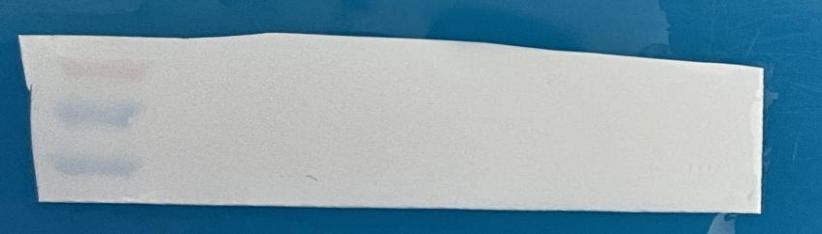

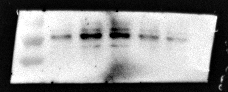

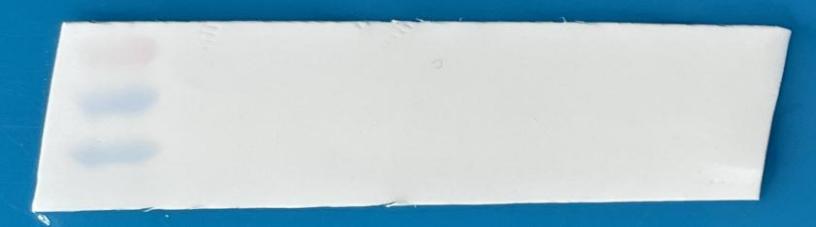


1

3

2

9.MMP13 (60kDa）

Emodin(μM) _- - 5 10 20_

H_2_O_2_ (mM) _- + + + +_

3

2

1


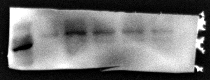

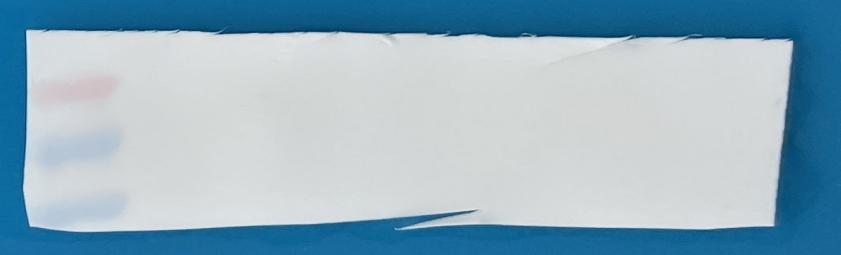

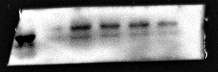

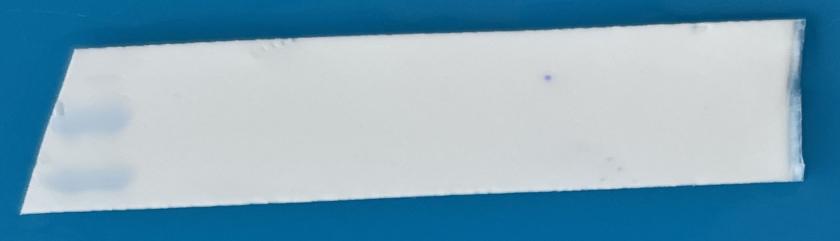

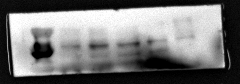

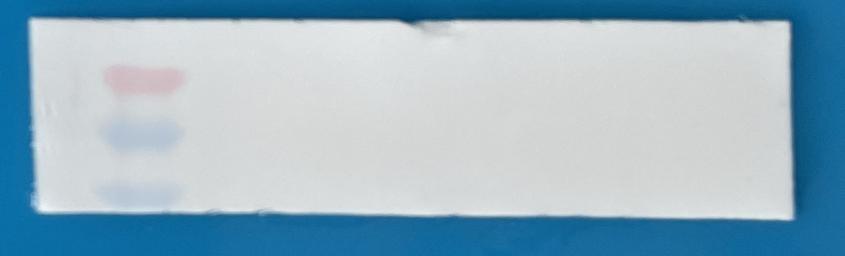


10.GAPDH (36kDa）

3

2

1


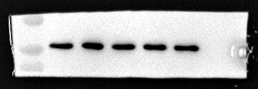

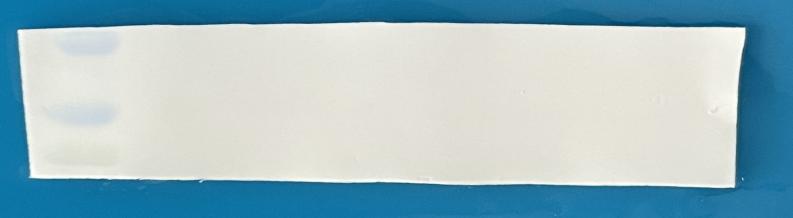

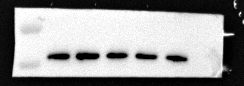

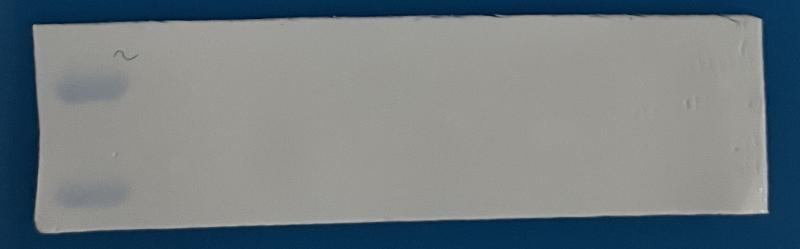

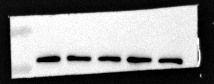

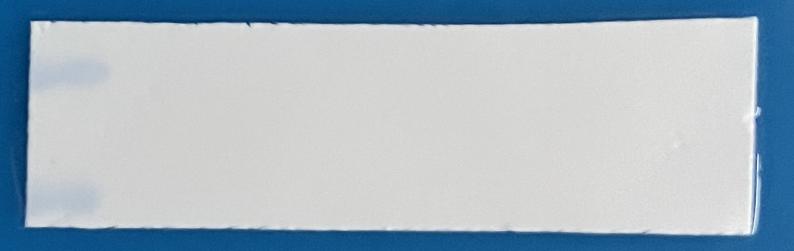


11.Lamin B1 (66kDa)


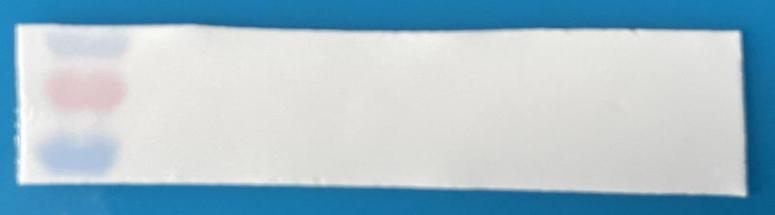

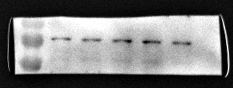

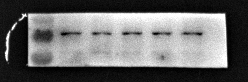

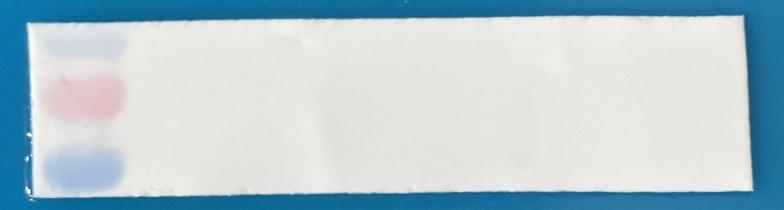

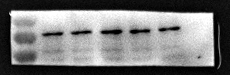

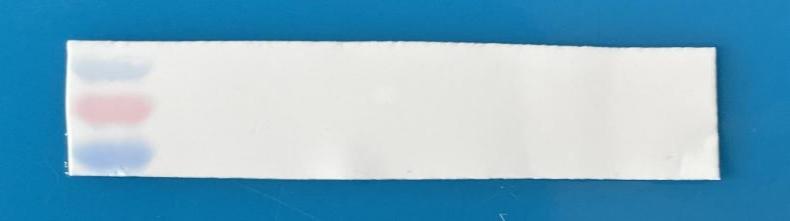


1

2

3
